# Methicillin-resistant and methicillin-susceptible *Staphylococcus aureus* in French hedgehogs admitted to a wildlife health center

**DOI:** 10.1016/j.onehlt.2024.100938

**Published:** 2024-11-19

**Authors:** Marisa Haenni, Séverine Murri, Caroline Lefrère, Jesper Larsen, Antoine Drapeau, Julie Botman, Pauline François, Philippe Gourlay, François Meurens, Jean-Yves Madec

**Affiliations:** a ANSES – Université de Lyon, Unité Antibiorésistance et Virulence Bactériennes, Lyon, France; b Oniris VetAgroBio, Nantes, France; c Department of Bacteria, Parasites & Fungi, Statens Serum Institut, Copenhagen, Denmark; d Oniris VetAgroBio, Centre Hospitalier Universitaire Vétérinaire, Centre Vétérinaire de la Faune Sauvage et des Ecosystèmes (Wildlife Health Centre), Nantes, France; e Oniris, INRAE, BIOEPAR, Nantes, France; f Centre de Recherche en Infectiologie Porcine et Avicole, Faculté de Médecine Vétérinaire, Université de Montréal, Saint-Hyacinthe, QC J2S2M2, Canada

**Keywords:** *mecC*, MRSA, Hedgehogs, France, *tst* gene

## Abstract

The *mecC* gene conferring methicillin-resistance has always been found on a SCC*mec* type XI element and is largely restricted to the few clonal complexes CC130, CC1943, CC425, CC49 and CC599. The occurrence of the *mecC* gene in many different hosts highlighted its One Health importance, even though European hedgehogs (*Erinaceus europaeus*) are considered its natural reservoir, most probably because of the selective pressure imposed by beta-lactam-producing dermatophytes (*Trichophyton erinacei*) that colonize the skin of these mammals. Surprisingly, while the presence of *T. erinacei* on the French territory has been proven, no *mecC*-positive methicillin-resistant *Staphylococcus aureus* (MRSA) isolate has been reported yet from hedgehogs. We thus sampled 139 hedgehogs brought to a wildlife center; 128 were *S. aureus* carriers and 25 (18.0 %) presented a MRSA isolate, of which 21 (15.1 %) displayed the *mecC* gene. All 161 *S. aureus* collected were whole-genome sequenced. The *mecC*-MRSA belonged to the classical CCs, i.e. CC130, CC1943 and CC49. The majority (98/139, 70.5 %) of the methicillin-susceptible *Staphylococcus aureus* (MSSA) isolates also belonged to these three CCs. A phylogenetic comparison with *mecC*-MRSA isolates from all over Europe and New-Zealand showed local adaptations, despite the fact that they all belonged to the same CCs. The acquisition of the SCC*mec* type XI element by a concomitant MSSA could not be observed in the same animal, but such a transfer might be suggested since identical clones were identified, one MSSA and one MRSA, though in different animals. In parallel, we conducted a detailed analysis of the SCC*mec* type XI element as well as specific virulence factors (a *tst* variant and the *vwb*_SaPI_ gene). Results led us to hypothesize that the *mecC* gene might be acquired through selective pressure of *T. erinacei* on MSSA, some of which were acquired a long time ago from ruminants and are now colonizing the skin of the hedgehogs.

## Highlights

• 18.0 % (25/139) of the hedgehogs presented at least one MRSA.
• Four animals were *mecA*-MRSA carriers, 21 were *mecC*-MRSA carriers.
• *mecC*-MRSA belonged to CC130/ST130, CC1943 (ST1943 and ST2361) and CC49/ST6834.
• 98/139 (70.5 %) MSSA isolates also belonged to CC130, CC1943 and CC49.
• The *sec*, *sel* and *tst* genes were observed in 8 MRSA CC1943 and 22 MSSA isolates.

## 1 Introduction

Methicillin-resistance in *Staphylococcus aureus* (MRSA) has long been attributed to the unique presence of the *mecA* gene, carried by different SCC*mec* elements. However, in 2011, a new methicillin-resistance gene, first called *mecA*_LGA251_ and finally named *mecC*, carried by the SCC*mec* type XI, was described in cattle and humans in the UK and Denmark [1]. Even though human and livestock-associated MRSA clones usually carry the *mecA* gene*, mecC*-MRSA have since then been found both in humans at a low prevalence and in a wide range of animals including goat, sheep, horses, companion animals and wild animals such as rats, hares, boar or deer [2]. This large distribution and the potential transmission events between hosts are highlighting the One Health dimension and importance of this gene. European hedgehogs (*Erinaceus europaeus*) are considered the primary natural reservoir of the *mecC* gene, probably acquired under the selection of the dermatophytes *Trichophyton erinacei* that colonize their skin and produce beta-lactams. Proportions of *mecC*-carriers in hedgehogs reached 65.9 % in England and Wales, 63.6 % in Sweden, 60.6 % in Denmark, 50.0 % in Czech Republic or 47.6 % in the Netherlands [[3], [4], [5]], while lower proportions were reported in Portugal (29.6 %), Finland (9.6 %), New Zealand (5.9 %) or Hungary (1.0 %) [3,6,7]. However, these percentages should be treated with caution due to differences in the methodology used in each study.

The *mecC* gene is strongly associated to clonal complexes (CC)130, CC1943 and CC425, and to a lesser extent to CC49 and CC599. *mecC*-MRSA usually carry the *blaZ* gene next to *mecC* but display no other gene conferring resistance to antibiotic families other than beta-lactams. The *mecC* gene has always been found located on an SSC*mec* type XI element, which was used by Larsen et al. [3] to estimate the temporal emergence of specific lineages among the three major CCs cited above. CC1943, split into three lineages, might date back 200 years and be the more ancient CC; CC130 emerged through 10 lineages that likely appeared between 1900 and 2000; finally, CC425 could be the last CC to appear, between 1940 and 2000 depending on the lineage.

The origin of *mecC*-MRSA is still debated. Larsen et al. characterized 149 MSSA isolates belonging to the same CCs as the *mecC*-MRSA, among which only 25 were collected from hedgehogs and belonged to CC49 (*n* = 13), CC130 (*n* = 11) and CC425 (n = 1) [3]. These methicillin-susceptible *S. aureus* (MSSA) constituting the hedgehogs' natural skin flora might thus be the recipients for the SCCmec type XI element. In Larsen's study, CC49/MSSA were only found in hedgehogs, while a significant number of CC130 and CC425 were also found in ruminants (cattle, goat, sheep) and wild animals (wild boar, red deer). In parallel, *mecC*-MRSA were rarely found in CC49 (*n* = 2 from New Zealand) but abundant in CC130 (*n* = 786) and CC425 (*n* = 76) isolates, leading to the hypothesis that CC130 and CC425 emerged from ruminants and wild animals before acquiring methicillin resistance.

So far in France, *mecC*-MRSA were only identified in humans, cattle and horses [8,9], and the nine French hedgehogs tested in Larsen's study were *mecC*-negative [3], despite the fact that 24.6 % of French hedgehogs were found to be carriers of *T. erinacei* [10]. This prompted us to perform an extensive sampling in hedgehogs, whose goal was (i) to estimate the prevalence of *mecC*-MRSA carried by hedgehogs on their skin, and (ii) to molecularly characterize both MSSA and MRSA found in these animals in order to try and understand the pathways by which the SCC*mec* type XI element was acquired.

## 2 Materials and methods

### 2.1 Sampling, bacterial isolation and identification

Between June 2020 and April 2021, 139 hedgehogs were sampled at the wildlife health center in Oniris VetAgroBio Nantes (France). Skin samples were taken using e-swabs (Biomerieux, France) by scrubbing the skin from the head to the rump, dorsally and ventrally, including potential zones presenting lesions. Swabs were kept at 4 °C for maximum 48 h before being plated on ChromID *S. aureus* and ChromID MRSA (BioMerieux) without any enrichment step.

Two PCRs targeting the 16 s rRNA, *mecA* and *nuc* genes as well as the *mecC* gene were performed to confirm the identification and to detect the gene responsible for methicillin-resistance [1,11].

### 2.2 Antimicrobial susceptibility Testing

Antimicrobial susceptibility testing was performed using the disk diffusion method and interpreted according to the guidelines of the Antibiogram Committee of the French Society for Microbiology (www.sfm-microbiologie.org). The protocol used and clinical breakpoints for antibiotics of veterinary interest are included in the French veterinary referential (last version 2023, https://www.sfm-microbiologie.org/2023/06/15/casfm-veterinaire-2023/). For antibiotics of human interest, clinical breakpoints of the EUCAST/CA-SFM referential were used (last version 2024, https://www.sfm-microbiologie.org/wp-content/uploads/2024/06/CASFM2024_V1.0.pdf). *S. aureus* ATCC 25923 was used as quality control. Fifteen antibiotics of veterinary and/or human interest were tested (Mast Diagnostics, France) (Table S1).

### 2.3 Whole genome sequencing using short-read Illumina

DNA was extracted using the NucleoSpin Microbial DNA extraction kit (Macherey-Nagel, France). Library preparation and short-read whole-genome sequencing using Illumina NovaSeq6000 technology was outsourced (Eurofins, Germany). Reads were quality trimmed, de novo assembled using Shovill v1.0.4 and the quality of assemblies was assessed using QUAST v5.0.2 (Table S2).

### 2.4 Genomic analyses

Typing was performed using MLST v2.0.9 and spaTyper (http://www.genomicepidemiology.org/). Clonal complexes (CCs) were identified by goeBURST (http://www.phyloviz.net). Identification of resistance and virulence genes was performed using Resfinder 4.1 and VirulenceFinder v2.0.3 with a minimum identity of 95 %. The *blaZ* genes were compared using Clustal Omega (v1.2.4) and clusters were visualized using iTOL v6.0 (http://itol.embl.de). The *vwb*_SaPI_ gene was searched by Blastn v2.5.0 using the reference sequence HM211303, with 90 % length and 70 % similarity. The chromosomal *vwb* gene was differentiated from the one carried by a SaPI by Clustal Omega using the strains and references published by Larsen et al. [3].

The cgMLST was determined (https://github.com/bvalot/pyMLST) using the schemes available on the www.cgmlst.org/ncs webpage (for allelic matrices, see Table S3). The analysis was performed on MSSA/MRSA isolates sequenced in this study (*n* = 161) and those reported from hedgehogs by Larsen et al. (see Larsen's Table S1 for accession numbers). Cut-offs for highly related strains were set at <10 allelic differences. Two isolates (MSSA #58301 and MRSA #58307) which presented identical cgMLST profiles were further analyzed using PPanGGOLiN v2.1.0. The presence of the SCC*mec* element (FR821779) was looked for in the two isolates. SNPs were inferred using Gubbins v3.3.5 and SNP-dists v0.8.2.

A SNP-based phylogeny was further constructed on all CC130, CC49 and CC1943 isolates using Roary as previously published ([12]; Roary v3.13.0, Gubbins v2.4.1 and snp-dists v0.7.0), each rooted with the ST425 reference strain NC_017349. Phylogenetic trees were visualized with iTOL.

### 2.5 Characterization of the SCC*mec* elements

The SCC*mec* element of the LGA251 strain was retrieved between the nucleotide positions 34402 and 63839 as indicated by Larsen et al. [3]. The SCC*mec* sequence was searched in all 344 *mecC*-MRSA from hedgehogs reported by Larsen et al. to build up a database of SCC*mec* variants comprising elements of >20°000 bp (*n* = 321) to avoid fragmented elements, the size of the SCC*mec* type XI element being around 30 kb [13]. Sequences were blasted against the 23 *mecC*-MRSA-positive isolates so that they could be assigned to a SCC*mec* variant. The tree was generated using a multiple alignment with Clustal Omega and visualized with iTOL.

### 2.6 Accession number

The project was deposited in GenBank under the BioProject accession number PRJNA1060918.

## 3 Results

### 3.1 Proportions of methicillin-resistant *S. aureus* and antimicrobial susceptibility

Among the 139 hedgehogs tested, 92.1 % (128/139) presented at least one *S. aureus* isolate, of which 25 (18.0 %) presented an MRSA isolate. Over the 128 animals displaying a *S. aureus* isolate on their skin, 30 were released in nature (Table S1), among which one was *mecA*-positive and five were *mecC*-MRSA-positive. One to three *S. aureus* were collected per animal, so that 161 non-duplicate isolates were sequenced. Two hedgehogs (#20–1318 and #21–0349, Table S1) each presented two different *mecC*-MRSA clones, leading to a collection of 27 MRSA carrying either the *mecA* (*n* = 4; 4/161, 2.5) or the *mecC* (*n* = 23; 14.3 %) gene.

### 3.2 Genetic diversity in *S. aureus* isolates

**Fig. S1:**
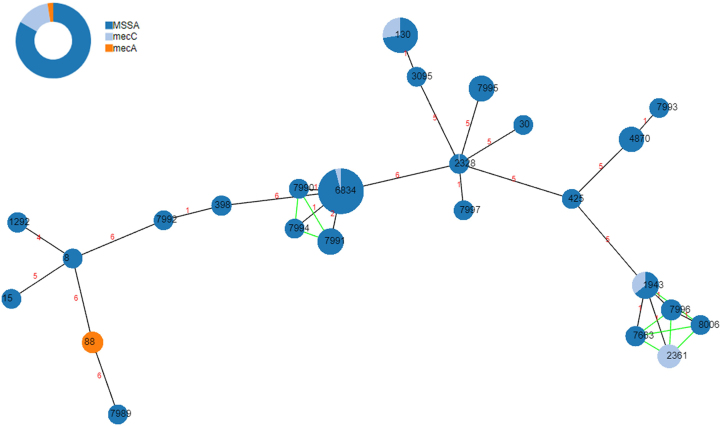
Clustering by clonal complexes (CCs) of the different sequence types (STs) found in this study. Analysis was performed using the goeBURST algorithm (phyloviz.net).

Twenty-five different STs comprising one (*n* = 13) to 48 isolates (n = 1) were identified (Table 1, Table S1). The four *mecA*-MRSA belonged to ST88 and were clonally related (2–6 allelic differences, Table S3). The *mecC*-MRSA belonged to CC130/ST130 (*n* = 8), CC1943/ST2361 (n = 8), CC1943/ST1943 (*n* = 5) and CC49/ST6834 (n = 2) (Fig. S1).

**Table 1:** Distribution of the *blaZ* and *mec* genes in the different clonal complexes among French hedgehogs.

Clonal complex (CC)	Associated sequence types (no of isolates)	Total	*bla*Z_LGA251_ + *mecC*	*blaZ*	*mecA*
		No of isolates (no of hedgehogs)			
CC1	1292 (2)	2 (2)	0 (0)	2 (2, *blaZ*_3)	0 (0)
CC8	8 (1)	1 (1)	0 (0)	0 (0)	0 (0)
CC15	15 (1)	1 (1)	0 (0)	1 (1, *blaZ*_4)	0 (0)
CC30	30 (1)	1 (1)	0 (0)	1 (1, *blaZ*_2)	0 (0)
CC49	6834 (48), 7990 (1), 7991 (13), 7994 (1)	63 (59)	2 (2, *blaZ*_10^b^)	13 (13, *blaZ*_10)	0 (0)
CC88	88 (4)	4 (4)	0 (0)	4 (4, *blaZ*_4)	4 (4)
CC130	130 (29), 3095 (1)	30 (27)	8 (8, *blaZ*_9^b^)	21 (21, *blaZ*_6)	0 (0)
CC398	398 (2), 7992 (1)	3 (3)	0 (0)	2 (2, *blaZ*_5)	0 (0)
CC425	42 (1)	1 (1)	0 (0)	1 (1, *blaZ*_6)	0 (0)
CC1943	1943 (14), 2361 (8), 7663 (2), 7996 (3), 8006 (1)	28 (26)	13 (12, *blaZ*_10^2^)	0 (0)	0 (0)
Others	2328 (1), 4870 (11), 7989 (1), 7993 (1), 7395 (12), 7997 (1)	27 (27)	0 (0)	1 (1, *blaZ*_1)	0 (0)
Total	161	161 (152^a^)	23 (22)	46 (46)	4 (4)

a The total number of hedgehogs indicated here exceeds the total number of hedgehogs tested since one animal could carry several different isolates.

b *blaZ*_9 and *blaZ*_10 are two variants of the *bla*Z_LGA251_.

The majority (98/139, 70.5 %) of the MSSA belonged to the same CCs/STs as *mecC*-MRSA, and the most frequently identified lineage was CC49-ST6834/t208 (*n* = 41) (Table S1). Two MSSA isolates belonged to the CC398 clone and one to its single-locus variant (SLV) ST7992. Inside a specific ST*/spa*-type, most isolates were not clonally related (>10 allelic differences) (Table S3).

In 16 *mecC*-MRSA-positive hedgehogs, one MSSA was concomitantly isolated. The MSSA and *mecC*-MRSA both belonged to the same ST in only four cases (three to ST130, one to CC1943/ST2361) but did not display the same *spa*-type. Only two ST1943/t15312 isolates from different animals, one *mecC*-MRSA (#58307) and one MSSA (#58301), shared exactly the same cgMLST profile. A deeper SNP analysis showed that they differed by 5 SNPs, and the the MSSA #58301 completely lacked the SCC*mec* element.

### 3.3 Phylogenomic analysis of *S. aureus* isolated from hedgehogs

**Fig. 1:**
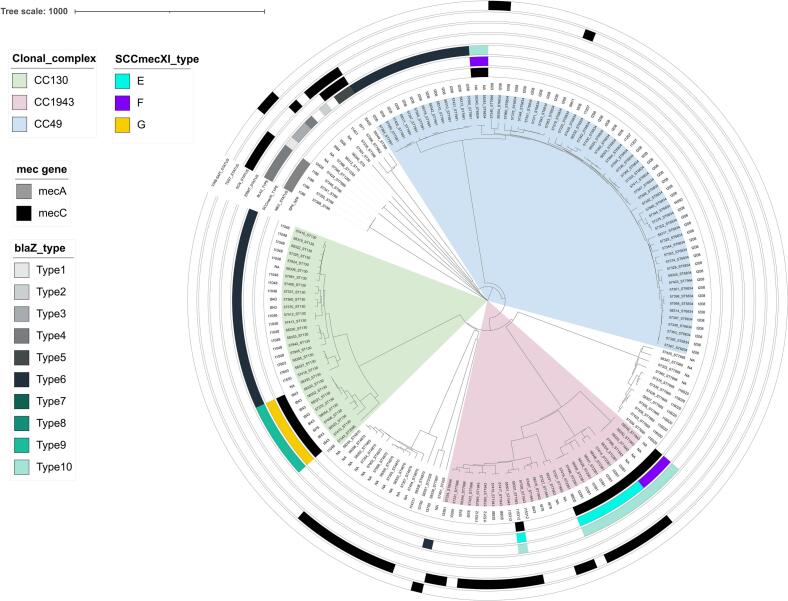
cgMLST-based phylogeny of the 161 French *S. aureus* isolates and additional metadata showing the presence/absence of the *mecA*/*mecC* and *ermT* genes, the *blaZ* types when present, the type of SCC*mec* element XI as described by Larsen et al. as well as the most relevant virulence genes (*scn*, *tst* variant and *vwb*_SaPI_).

The phylogenetic analysis showed that *mecC*-MRSA mostly clustered according to their *spa*-types, and separately from the *mecC*-negative isolates from the same ST (Fig. 1). All eight ST130 *mecC*-MRSA presented the *spa*-type t843, which was only identified in 2/21 MSSA ST130 (Table S1). Among CC1943, ST2361 isolates were all *mecC*-MRSA, while ST1943 isolates belonged to a non-typable *spa*-type (*n* = 4) and to t15312 (*n* = 1), the latter being also identified in four MSSA. Finally, the two *mecC*-MRSA-positive CC49/ST6834 isolates presented a non-typable *spa*-type, which was not identified among the 46 remaining CC49-MSSA.

**Fig. 2:**
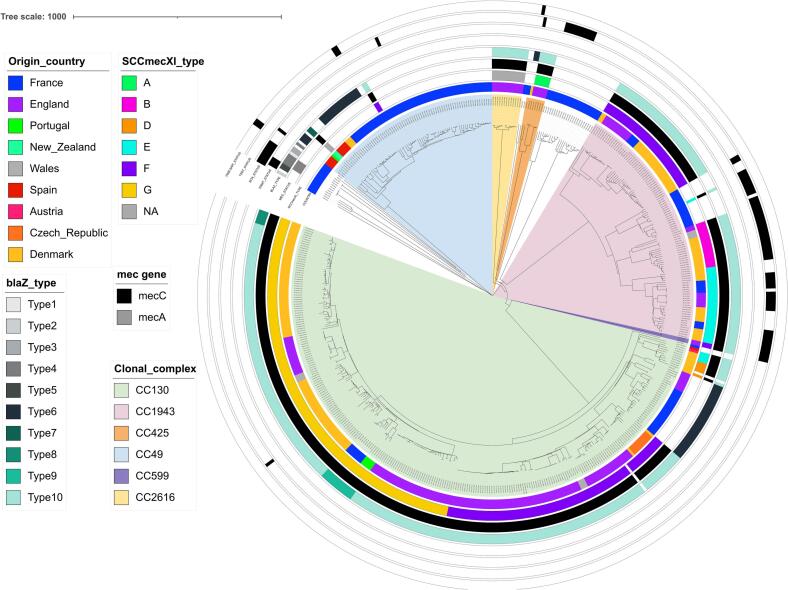
cgMLST-based phylogeny of the 161 French *S. aureus* isolates compared to the 344 *S. aureus* reported by Larsen et al.. Additional metadata are showing the country of origin, the presence/absence of the *mecA*/*mecC* and *ermT* genes, the *blaZ* types when present, the type of SCC*mec* element as described by Larsen et al. as well as the most relevant virulence genes (*scn*, *tst* variant and *vwb*_SaPI_).

**Fig. S2:**
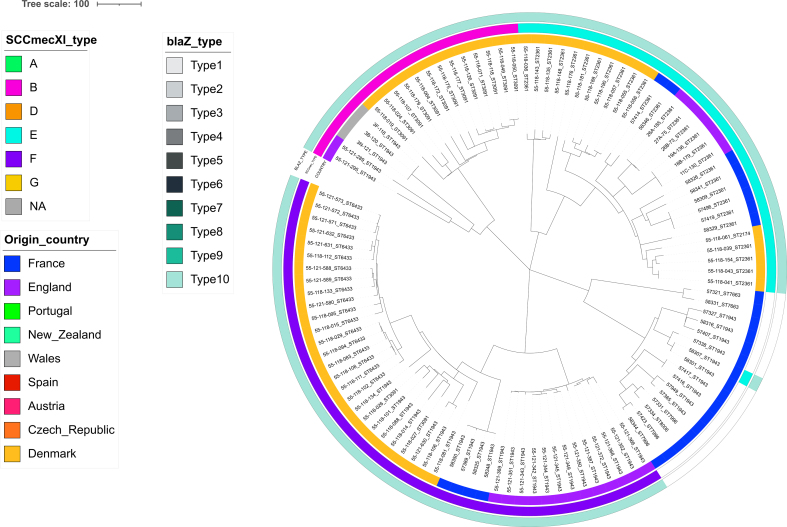
SNP-based analysis of the CC1943 isolates (*n* = 109), using the reference NC_17349 to root the tree.

**Fig. S3:**
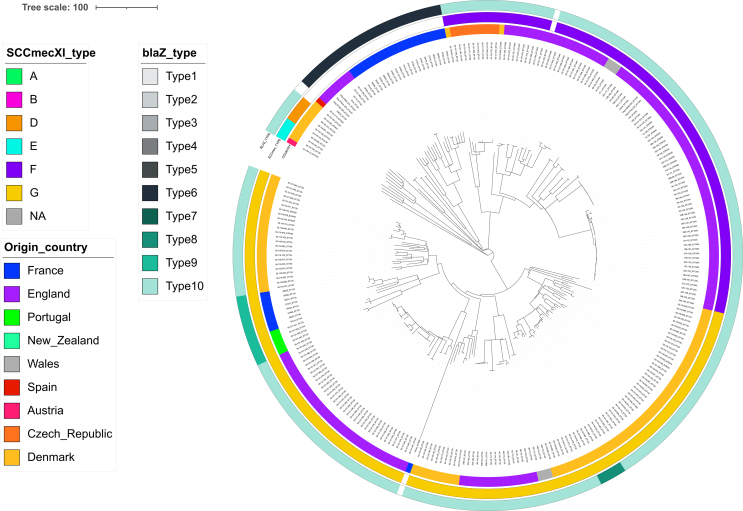
SNP-based analysis of the CC130 isolates (*n* = 283), using the reference NC_17349 to root the tree.

**Fig. S4:**
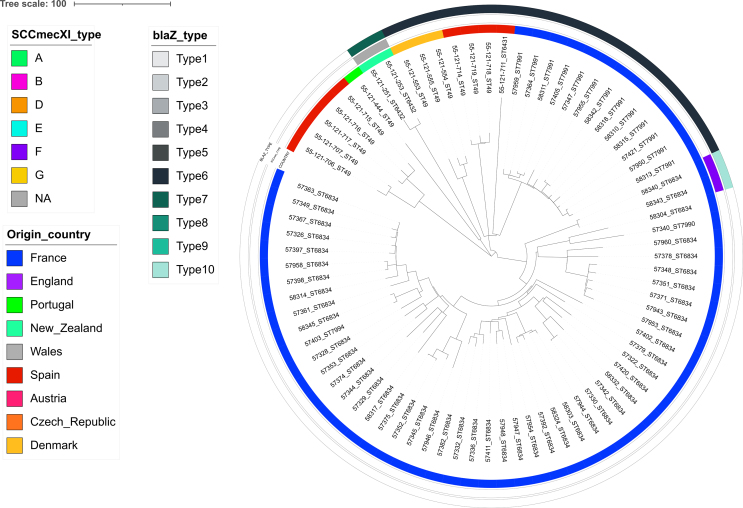
SNP-based analysis of the CC49 isolates (*n* = 79), using the reference NC_17349 to root the tree.

The 161 *S. aureus* isolated from French hedgehogs were further compared with the 344 *S. aureus* reported by Larsen et al., among which 25 were MSSA and 344 *mecC*-MRSA (Fig. 2). Overall, isolates clustered by country. Nevertheless, all eight CC1943/ST2361 isolates from this study individually shared similarities (75–96 allelic differences) with five isolates collected in 2016 in Jutland, Denmark (Table S3). Most of the CCs identified were reported at least from two countries, except for CC2616 (ST6460) that was only found in *mecC*-MRSA from England. ST4870 and ST7995, belonging to unassigned CCs, were only found in French MSSA. A refined SNP analysis of CC1943 (Fig. S2) confirmed their genetic similarity, as well as the clustering by country for CC130 and CC49 (Fig. S3 and S4). This analysis also showed that *mecC*-MRSA evolved differently from the MSSA belonging to the same CC.

### 3.4 Analysis of the SCC*mec* XI elements

**Fig. S5:**
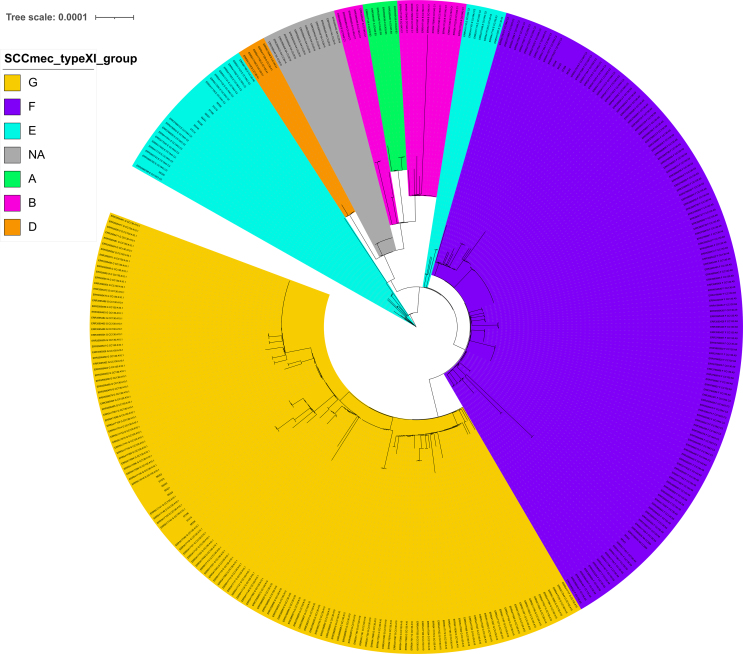
Typing of the SCC*mec* type XI element according to Larsen et al.

The type XI SCC*mec* elements of the 23 *mecC*-MRSA-positive isolates from this study were aligned with the 344 type XI SCC*mec* elements reported by Larsen et al. and assigned to different variants (Fig. S5). The eight CC1943/ST2361 and the unique ST1943/t15312 clustered with the CC1943-C2 lineage belonging to the E variant defined by Larsen et al., while the four remaining ST1943 presenting a non-typable *spa*-type clustered with the CC1943-C3 lineage belonging to the F variant. All eight ST130 isolates clustered with the CC130-A10 lineage from the G variant.

### 3.5 Antimicrobial resistance phenotypes and genotypes

**Fig. S6:**
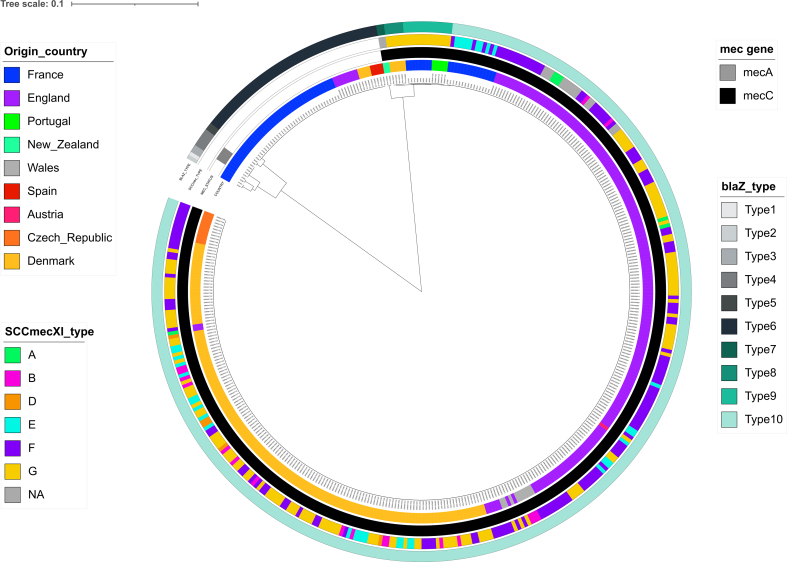
Clustal omega analysis of the *blaZ* genes identified in this study and in the one by Larsen et al.

Antibiograms revealed susceptibility to all antibiotics tested in 91 (91/161, 56.5 %) isolates. Resistance to macrolides/lincosamides conferred by the *ermT* gene was observed in the three CC398 isolates (1.9 %, of which two also presented the *blaZ* gene), while resistance to penicillin G was found in 69 isolates (42.9 %, including MRSA isolates). Ten different variants of the *blaZ* gene were identified among our isolates and those from Larsen et al. [3] (Fig. S6, Table S1). All *mecC*-MRSA displayed either the prototypic *blaZ*_LGA251_ gene (called *blaZ*_10, Fig. S6) or one of the two variants observed among CC130 isolates: *blaZ*_8 observed in five isolates from Denmark, and *blaZ*_9 present in 13 isolates (eight from France, five from Portugal). All penicillin-resistant MSSA isolates belonging to CC49, CC1943 and CC130 carried the *blaZ*_6 variant, while all isolates that did not belong to hedgehog-related CCs presented different *blaZ* variants (*blaZ*_1 to *blaZ*_5) (Table S1).

### 3.6 Virulence genes in MSSA and MRSA isolates

The immune evasion cluster (IEC) was identified in five isolates (four *mecA*-positive ST88 and one MSSA ST30 isolate) not related to hedgehogs' CCs/STs (Table S1). Thirty (18.6 %) isolates presented variants of the toxic shock syndrome gene *tst* (Fig. 2, Table S1). One variant, identical to a published sequence (Genebank: WP_001035595), was found in the unique #57379 isolate belonging to the CC49/ST6834/t208. The second variant was found in 10 isolates mostly belonging to CC1943/ST1943 (7/10, including one *mecC*-MRSA). Finally, the third variant, which was identified in 19 isolates including 8/10 MSSA ST4870 (unassigned CC) and all seven CC1943/ST2361/t3391 *mecC*-MRSA, was identical to a human strain isolated in the Netherland (HDE6524069) in 2020. All *tst*-positive isolates co-carried the enterotoxin *sec* and *sel* genes on the same contig. In Larsen's isolates, a *tst* gene was also found 33 isolates (33/344, 9.6 %), among which 30 belonged to the CC1943 (30/80, 37.5 %), one belonged to CC130 and two to CC599 (Fig. 2). The *vwb*_SaPI_ gene was identified in five isolates (Fig. 1, Table S1), including the only CC425 from this study.

## 4 Discussion

In this study, 18.0 % (25/139) of the hedgehogs presented at least one MRSA isolate. Four animals (2.9 %) presented a *mecA*-carrying ST88 isolate, a ST usually associated to community-acquired human MRSA, but that has also been reported in retail food in Asia or pigs in Africa [14,15]. All remaining 21 animals (15.1 %) were *mecC*-MRSA-positive, and the *mecC* gene was even found in two different *S. aureus* clones in two hedgehogs. The prevalence of the *mecC* gene was lower than what has been reported in UK (65.9 %), Sweden (63.6 %), Denmark (60.6 %), Czech Republic (50.0 %), the Netherlands (47.6 %) or Portugal (29.6 %), but higher than in Finland (9.6 %), New Zealand (5.9 %) or Hungary (1.0 %) [[3], [4], [5], [6], [7]]. These proportions should be taken with caution since they are surely influenced by methodological specificities (such as the presence or absence of an enrichment step, as well as the total number of animals tested) and might also depend on the prevalence of the *T. erinacei* dermatophyte. But, in any case, *mecC*-MRSA are surely not restricted to Northern Europe. They can be found wherever European hedgehogs are present, either in their natural habitat (from Southern Scandinavia and Finland to Western Europe), or in New Zealand where they were introduced in the late 1800s [16].

Hedgehogs studied here were sampled on arrival to the wildlife health center, so that contamination in the center is very unlikely. Bengtsson et al. also observed a high prevalence of *mecC*-MRSA carriers arriving at rehabilitation centers and thus suggested that the high prevalence of *mecC*-MRSA in Sweden might be partially due to the release of *mecC*-MRSA-positive animals [5]. This hypothesis has been ruled out as similarly high proportions of *mecC* carriers were reported in wild hedgehogs [17,18], but the question of releasing MRSA-positive animals remains. Here, 22.2 % of the hedgehogs were brought back to nature, among which one was *mecA*-positive and five were *mecC*-MRSA-positive when they arrived in the center. This should prompt veterinarians and people in contact to take precautions, first because *mecC*-MRSA are zoonotic pathogens, and second to avoid dissemination among animals from the rescue center, which are all intended to be released.

The CCs in which the *mecC* gene was identified were CC130/ST130, CC1943 (ST1943 and ST2361) and CC49/ST6834. This is in line with international reports and with the few *mecC*-MRSA collected from both humans and animals in France, which all belonged to CC130, except one ST49 (CC49) from a horse [3,8,9,19]. Interestingly, 98/139 (70.5 %) MSSA isolates identified here also belonged to these three CCs. We might thus hypothesize that these CCs are part of the normal flora of hedgehogs, and that the SCC*mec* type XI element was selected and maintained in these dominant isolates which then, according to the SNP-based phylogeny, evolved differently from the susceptible strains. In our collection, two identical CC1943 isolates were identified, one carrying the *mecC* gene and the other not, suggesting the acquisition of the SCC*mec* element by an MSSA isolate. Even though the loss of this element by a *mecC*-MRSA cannot be ruled out, the hypothesis of a recent acquisition might be favoured since the *mecC*-positive-MRSA clustered among the MSSA isolates and not with the other *mecC*-MRSA that evolved differently. Nevertheless, none of the *mecC*-MRSA-positive hedgehogs described here concomitantly carried an identical *mecC*-negative isolate, and the *mecC* gene was mostly found in isolates belonging to a different *spa*-type than their MSSA counterparts. Consequently, further studies are needed to understand whether these ST/*spa* types are originating from the hedgehogs themselves or, as also hypothesised, from other animals in contact, such as ruminants or wild animals that are also carriers of MSSA CC130 and CC425 [3].

Larsen et al. estimated when the different *mecC*-MRSA lineages emerged [3]. In our study, 9/23 (39.1 %) of the hedgehog isolates belonged to the most ancient linage CC1943-C2 dating back 200 years, and four (17.4 %) belonged to the CC1943-C3 lineage that appeared in the 1900s. Interestingly, all but one CC1943-C2 isolates co-carried a variant of the toxic shock syndrome toxin *tst* gene, which was absent in all remaining *mecC*-MRSA from this study. In parallel, 22 of the MSSA isolates carried *tst* variants, of which 11 belonged to the CC1943 and 10 to the ST4870 (unassigned CC). All *tst*-positive isolates also carried the *sec* and *sel* genes coding for enterotoxins. In Larsen's isolates, 33 *tst*-positive isolates were also found, which were mostly distributed among the most ancient *mecC*-MRSA-positive isolates, i.e. in the CC1943-C1 (*n* = 18) – which was absent from the French isolates – and CC1943-C2 (*n* = 12) lineages. The presence of the *sec*, *sel* and *tst* genes indicated the presence of the SaPIbov described by Fitzgerald et al. [20]. This SaPIbov pathogenicity island has been frequently identified in bovine, sheep and goat [21], thus reinforcing the hypothesis of a ruminant source or common origin of these isolates. The *vwb*_SaPI_ gene, coding for a protein with coagulase activity against ruminant plasma, was found in five isolates of this study, including two CC49 and one CC425 MSSA. Larsen et al. identified this gene in numerous MSSA CC130 and CC425 from small ruminants across Europe, but in none of their hedgehogs. Our finding thus further emphasizes the hypothesis of a ruminant origin of these hedgehogs-associated MSSA.

## 5 Conclusion

A significant prevalence (25/139, 18.0 %) of MRSA in hedgehogs was observed in the north-west part of France, with a majority of animals (21/25) presenting the *mecC* gene. The CCs in which the *mecC*-MRSA were identified (CC130, CC1943, CC49) were also those found in 70.5 % of the MSSA isolates that massively colonized or infected hedgehogs. The most ancient lineage of *mecC*-MRSA-positive isolates, namely CC1943-C2, presented the SaPIbov element (*sec*, *sel* and *tst* genes), while five of our isolates carried the *vwb*_SaPI_ gene, which are both virulence factors frequently found in ruminant isolates. Our results suggest that *mecC*-MRSA-positive isolates might have emerged, under the pressure of dermatophytes, through the acquisition of the SCC*mec* type XI element by strains belonging to the dominant staphylococcal flora of hedgehogs. Altogether, this study reaffirms the One Health dimension of the spread of the *mecC* gene, connecting the natural reservoir (hedgehog) of *mecC*-MRSA with numerous other animal and human hosts through the dissemination of either *mecC*-negative or *mecC*-positive *S. aureus* lineages.

The following are the supplementary data related to this article.

Supplementary Table S1 Supplementary Table S1

Supplementary Table S2 Supplementary Table S2

Supplementary Table S3 Supplementary Table S3

## Funding

This research was funded by institutional support from 10.13039/501100007546ANSES.

## Transparency declarations

None to declare.

## Ethical approval

No ehtical approval was needed since only non-invasive swabs were taken upon arrival at the wildlife rescue center.

## CRediT authorship contribution statement

**Marisa Haenni:** Writing – review & editing, Writing – original draft, Funding acquisition, Formal analysis, Conceptualization. **Séverine Murri:** Writing – review & editing, Project administration, Methodology, Investigation. **Caroline Lefrère:** Writing – review & editing, Methodology, Investigation. **Jesper Larsen:** Writing – review & editing, Writing – original draft, Methodology, Formal analysis. **Antoine Drapeau:** Writing – review & editing, Software, Investigation. **Julie Botman:** Writing – review & editing, Investigation. **Pauline François:** Writing – review & editing, Software, Investigation, Data curation. **Philippe Gourlay:** Writing – review & editing, Supervision, Formal analysis, Conceptualization. **François Meurens:** Writing – review & editing, Supervision, Formal analysis, Conceptualization. **Jean-Yves Madec:** Writing – review & editing, Supervision, Funding acquisition.

## Declaration of competing interest

The authors declare that they have no known competing financial interests or personal relationships that could have appeared to influence the work reported in this paper.

## Data Availability

Data will be made available on request.
